# Brain Abscess From Dental Bacteria: A Case Report

**DOI:** 10.7759/cureus.87768

**Published:** 2025-07-12

**Authors:** Magdalena Staniszewska, Kamil Krzesniak, Pawel Kolasa, Beata Smielak

**Affiliations:** 1 Department of Neurosurgery and Cancer of the Nervous System, Regional Multi-Specialist Center for Oncology and Traumatology, Nicolaus Copernicus Memorial Hospital, Lodz, POL; 2 Department of Dental Technology, Medical University of Lodz, Lodz, POL; 3 Department of Prosthodontics, Medical University of Lodz, Lodz, POL

**Keywords:** dental infections, fusobacterium, odontogenic brain abscess, purulent infection, streptococcus constellatus

## Abstract

A brain abscess (BA) is a purulent infection of the central nervous system and can be associated with dental procedures. The paper presents a case of a patient diagnosed with an odontogenic BA. On admission, the patient was awake, alert, and oriented (Glasgow Coma Scale 15). The patient reported undergoing excision and drainage of an abscess in the submandibular area three years earlier. An MRI of the head showed a mass lesion (34x19x25 mm) located in the right parieto-occipital region, and an abscess was suspected. During the procedure, puncture and evacuation of the contents of the abscess in the right parieto-occipital area were performed. Oral microorganisms, including *Streptococcus constellatus *and *Fusobacterium gonidiaformans*, were isolated from the brain pus. A thorough radiographic and dental examination should be conducted during hospitalization in these patients. Always evaluate for periapical pathology in unexplained BAs, even in the absence of oral symptoms.

## Introduction

A brain abscess (BA) is a purulent infection of the central nervous system, visible as a sharply demarcated collection of pus surrounded by a vascularized capsule. In the USA, there are 1,500 to 2,500 cases per year, which gives an incidence of 0.3-1.3 cases per 100,000 inhabitants. Mortality ranges from 5 to 20%, with men aged 30-40 being affected two to three times as often as women [1,2]. Abscess formation is more common in people with weakened immunity, e.g., in cases of HIV infection or after chemotherapy, immunosuppression, or steroid therapy. BAs can also occur as a complication of surgical procedures, such as dental procedures or open head trauma [3]. The lesion can also occur following the accumulation of infected material from a submucosal abscess, a strong inflammatory process in the periapical tissues of the tooth. Nevertheless, the main cause of BA is bacterial infection. Currently, the most common way of spreading infection is via the blood; however, in 10-60% of cases, the source of infection cannot be identified [2]. Until 1980, the most common route of infection to the brain was through continuity, i.e., directly from a nearby focus of infection. Such spread most often originates from the middle ear, paranasal sinuses, and teeth [3]. Odontogenic infections can be spread both by continuity and hematogenously, and originate mainly from the molars. They can also be associated with dental procedures, typically performed within four weeks of the appearance of the BA [4]. The most common causes are periapical tissue inflammation and periodontitis. Even mild and extensive periodontal diseases, trauma without bleeding, and tooth mobility in the socket often cause sinusitis and bacteremia [5,6]. Oral pathologies often result from poor hygiene practices [3-6]. Dental abscesses are usually located in the frontal lobe and less frequently in the temporal lobe; however, hematogenous dissemination typically leads to the formation of multiple multi-chamber abscesses of the brain (10-50%) [6].

In up to 70% of cases (combined data), the bacteria isolated from BAs are aerobic, anaerobic, and microaerophilic streptococci (i.e., requiring oxygen, but in a concentration lower than those found in the atmosphere) [5]. These include streptococci, *Streptococcus anginosus* (milleri), found in the mouth, appendix, and genital tract. In addition, *Staphylococcus aureus* is found in 10-20% of cases, usually following head trauma or infective endocarditis; 23-33% harbor Gram-negative bacilli (*Proteus sp., Escherichia coli, Klebsiella sp., Pseudomonas aeruginosa, *and* Enterobacter sp.*), usually those with otitis media, sepsis, or immunodeficiency or after neurosurgery; and 20-40% exhibit anaerobes (*Bacteroides* and *Prevotella sp.*) [3,6]. An odontogenic source may be associated with *Actinomyces* infection [4]. However, sterile cultures are found in 0-43% of cases, most often after prior antibiotic therapy [3]. In people with impaired immunity, e.g., in patients receiving immunosuppression or AIDS patients, BAs may be the result of infection with *Toxoplasma gondii, Nocardia asteroides, Candida albicans, Listeria monocytogenes, Mycobacterium, *or* Aspergillus fumigatus*, with fungal infections prevailing [2].

There are classically four stages of infection leading to abscess formation: early encephalitis, usually lasting one to three days, characterized by tissue necrosis with neutrophil infiltration and increasing edema; late encephalitis, occurring between days four and nine, associated with macrophage and lymphocyte infiltrates; and early (from day 10 to 13) and late abscess capsule formation (from day 14) with the presence of infiltration of plasma cells and myofibroblasts [3]. However, the individual stages vary considerably depending on the type of pathogen. In anaerobic infection, it is impossible to distinguish the successive phases of capsule formation, and encystment occurs very late [1,5]. Clinical symptoms depend on the size of the abscess, its location, the virulence of the pathogen, and the immune status of the patient [7]. The presence of immune disorders may mask symptoms. Headache is the most common symptom in 70-75% of patients. Sudden intensification of pain accompanied by meningeal symptoms and deterioration of the general condition may indicate the penetration of the abscess into the ventricular system [8,9]. Fever occurs in only half of patients; hence, the classic triad of increased body temperature, headache, and focal symptoms is relatively rare. As the mass grows, irritating the brain and increasing intracranial pressure, the following symptoms appear: nausea, vomiting, epileptic seizures, and disturbances of consciousness. The clinical condition typically deteriorates more quickly than for neoplasms. The nature of epileptic seizures and focal symptoms depends on the location of the abscess.

The diagnostic test of choice is magnetic resonance imaging (MRI), which offers greater accuracy than computed tomography (CT); it also enables early detection of encephalitis and better visualization of dissemination to the ventricles and subarachnoid space and the presence of satellite lesions [10]. Currently, the blood-borne route is the most common cause of infection spread. It should be emphasized that before the era of computed tomography and MRI, the mortality rate of patients with BA was 40-60%. Contrast-enhanced diffusion-weighted MRI is the gold standard for diagnosing BA due to its high accuracy, sensitivity, and ability to differentiate from other focal lesions. CT can be used as an initial study, especially in an emergency setting, but it does not replace a full evaluation with an MRI. Currently, the mortality rate is around 10%, while more than 50% of cured patients present with persistent neurological deficits and require antiseizure treatment. Laboratory tests typically reveal increased erythrocyte sedimentation rate (ESR) and C-reactive protein (CRP). Slight leukocytosis is observed in 60-70% of cases [2]. Blood should be collected for culture, although the result rarely allows for identification of the pathogen. Elevated opening pressure is usually found during lumbar puncture, and higher leukocyte and protein levels are noted in the cerebrospinal fluid. It is rare to isolate the pathogen from the cerebrospinal fluid (6-22%), unless the abscess penetrates the ventricular system [3]. Lumbar puncture is rarely performed due to the risk of impaction, especially in large lesions [2]. Alternatively, stereotactic, neuronavigated, or ultrasound-guided biopsy can be performed. However, it should be noted that up to 30% of patients who received antibiotics before biopsy will have sterile cultures [10]. If a bacterial abscess is suspected and the culture shows no growth, 16S ribosomal RNA amplification and sequencing may help identify the pathogen [11,12].

Antibiotic therapy alone brings the best results in small (< 25 mm) non-encapsulated abscesses in patients who show clinical improvement within the first week of treatment. Conservative treatment is also recommended for patients in very severe conditions, i.e., with multiple, small abscesses, perhaps located in hard-to-reach structures, i.e., the brainstem, or in patients with concomitant encephalitis [2]. The indication for surgical treatment is the occurrence of the so-called mass effect, symptoms of increased intracranial pressure, the presence of an abscess in the vicinity of the ventricular system (due to the risk of its penetration), post-traumatic or postoperative abscesses that may arise because of leaving foreign material, abscesses of fungal origin, and a lack of improvement after conservative treatment. The recommended method of surgical treatment is removal of the abscess by puncture and aspiration of its contents. In the case of multiple abscesses, the largest one is selected for puncture. Unfortunately, more than 70% of patients require this procedure to be repeated due to the regrowth of the lesion. Sometimes it is necessary to remove the abscess together with the capsule by craniotomy [2,3]. In the case of suspected bacterial abscess, it is advisable to start therapy with broad-spectrum antibiotics after obtaining material for microbiological examination. In the case of *Staphylococcus aureus* infection, a third-generation cephalosporin should be administered with metronidazole; vancomycin can also be added after obtaining an antibiogram [12]. In the case of Gram-negative bacilli, such as *P. aeruginosa*, ceftazidime, cefepime, or meropenem are recommended. In the absence of any information as to the pathogen, it is advisable to start treatment with vancomycin, metronidazole, and cephalosporin.

## Case presentation

**Figure 1:**
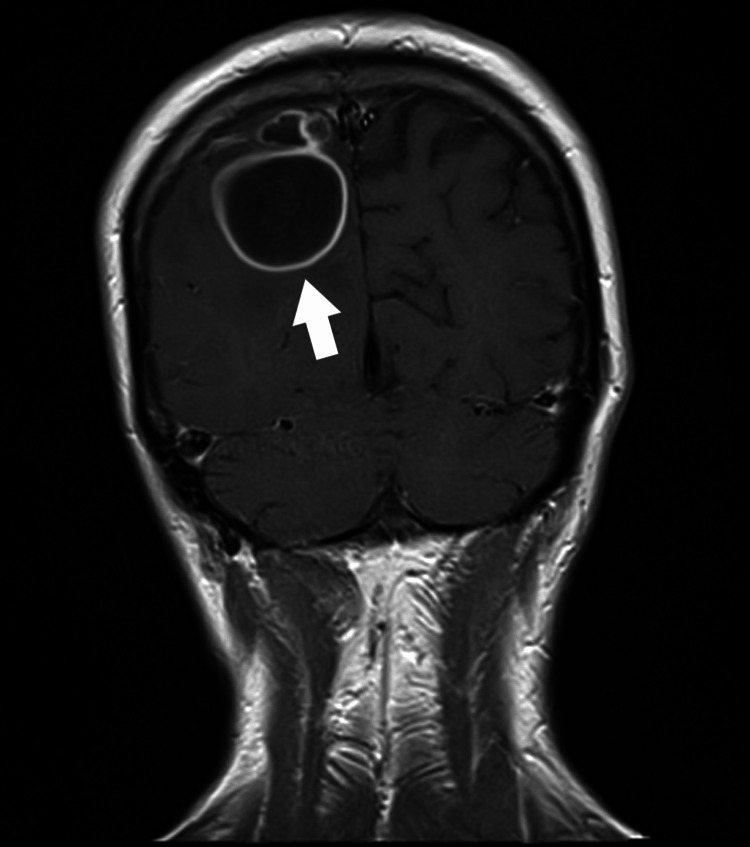
A follow-up MRI scan of the head revealed a residual abscess located in the right parieto-occipital region: anterior-posterior view (marked with a white arrow).

The paper presents a case of a patient diagnosed with an odontogenic BA. A 31-year-old Caucasian patient was referred to the Department of Neurosurgery with increasing left hemiparesis and motor coordination disorders. On admission, the patient was awake, alert, and oriented (Glasgow Coma Scale (GCS) 15). The patient reported undergoing excision and drainage of an abscess in the submandibular area three years earlier in the Department of Maxillofacial Surgery. The physical examination revealed the following: left-sided paresis with increased muscle tone (3/5 according to Lovett), impaired superficial sensation in the forearm and left lower leg, dysmetria, and visual field limitation in the upper and lower temporal quadrants of the left eye. An MRI of the brain taken on the 17th of February 2017 revealed a tumor located in the right parieto-occipital region, and an abscess was suspected (Figure 1).

The patient was qualified for surgical treatment. During the procedure, the abscess in the right parieto-occipital area was punctured and the contents evacuated. After the procedure, the patient was in generally good condition, awake, alert, and oriented (GCS 15). The physical examination revealed a decrease in left hemiparesis (4/5) and an increase in the visual field. A follow-up CT scan of the head revealed a residual abscess and postoperative changes (34x19x25 mm). *Streptococcus constellatus* and *Fusobacterium gonidiaformans* were cultured from the abscess material; a suspicion of odontogenic spreading infection was raised. Metronidazole and vancomycin were started, and after consultation with the maxillofacial surgeon, clindamycin was added.

**Figure 2:**
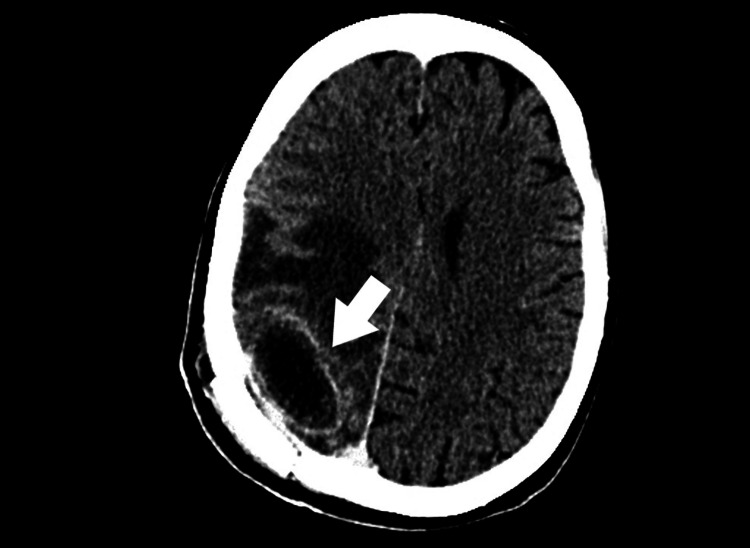
A follow-up CT scan of the head revealed a residual abscess at the surgical site with accompanying edema: a top view (25.02.2017) (marked with a white arrow).

On the 12th postoperative day, the clinical condition deteriorated. The patient was conscious and in verbal and logical contact, psychomotor slowed down, lying down, with intensified left-sided paresis, and no fever. She experienced a seizure, but without loss of consciousness. A follow-up contrast-enhanced CT of the brain showed an encapsulated abscess at the site of the previous surgical procedure with accompanying edema (Figure 2).

**Figure 3:**
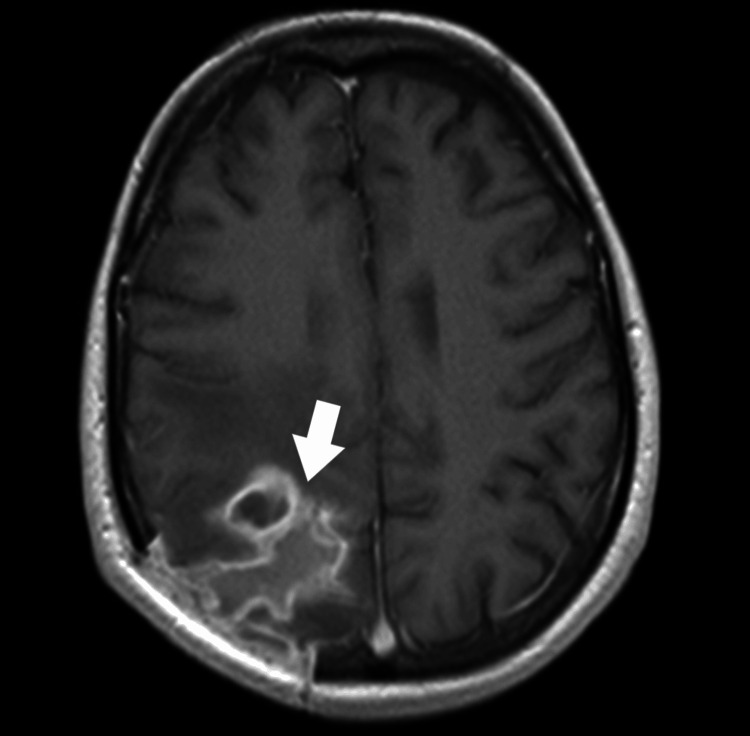
A follow-up MRI scan of the head revealed a postoperative lesion and possibly organizing suppuration: a top view (marked with a white arrow).

The patient was reoperated on. A right-sided parieto-occipital craniotomy was performed; the abscess was removed along with the capsule. Oral microorganisms, including *Streptococcus constellatus* and *Fusobacterium gonidiaformans*, were isolated from the brain pus. The identification of bacteria was performed using the automatic matrix-assisted laser desorption ionization-time of flight (MALDI-TOF) method. CSF culture was negative. After the procedure, the patient was conscious and in verbal and logical contact. Physical examination revealed left hemiparesis (2-3/5), which was more pronounced in the left upper limb. The patient underwent rehabilitation with improvements. The patient was awake, alert, and oriented. After rehabilitation, physical examination revealed left hemiparesis (4/5). After consultation with the oral and maxillofacial surgeon, the patient was qualified to remove tooth 27, which could be the potential source of infection. The tooth had radiographic periapical changes (CT) and was removed. An MRI of the brain performed after the second surgery revealed postoperative lesions and possibly an organizing abscess (Figure 3).

**Figure 4:**
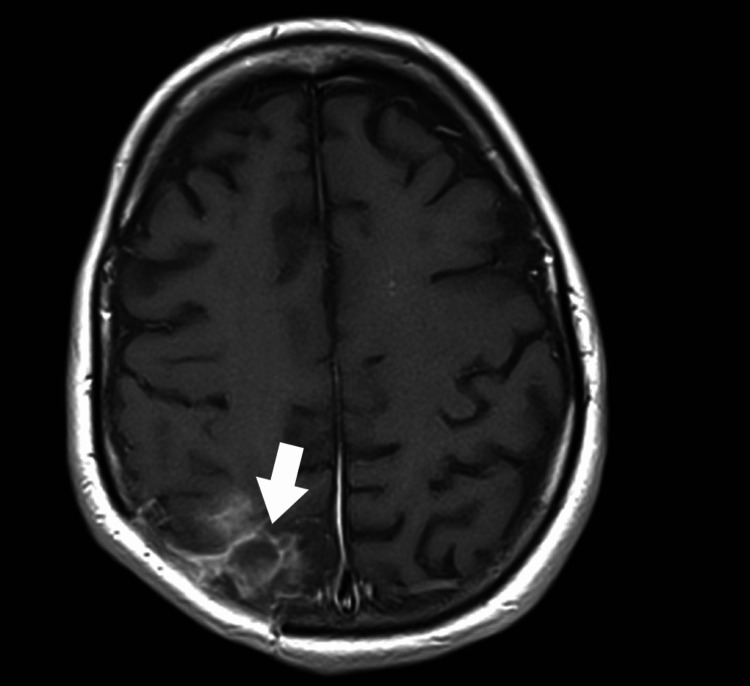
A follow-up MRI scan of the head revealed a residual postoperative change located in the right parieto-occipital area: a top view (marked with a white arrow).

Subsequent imaging studies indicated stabilization of changes with a tendency to regression. The patient was discharged home with a recommendation for further care at the Neurosurgical Outpatient Clinic and continued antiepileptic treatment. Five months after the surgical treatment, a follow-up MRI of the brain was performed (Figure 4).

**Figure 5:**
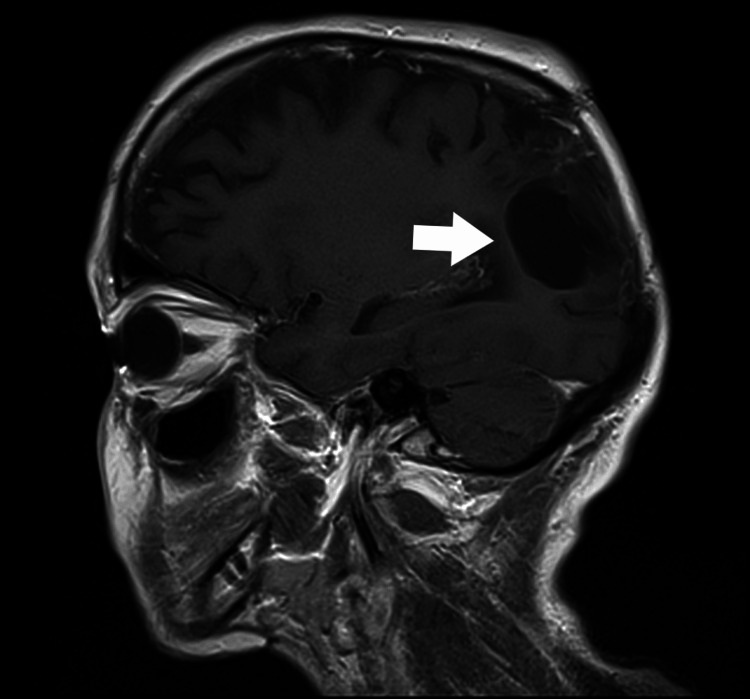
A follow-up MRI scan of the head 1.5 years after surgery: a side view. No signs of recurrence of the abscess (marked with a white arrow).

Imaging studies showed no signs of recurrence. The patient was found to be stable during a follow-up visit 18 months after the surgical treatment. MRI of the brain showed no signs of recurrence of the abscess (Figure 5).

**Figure 6:**
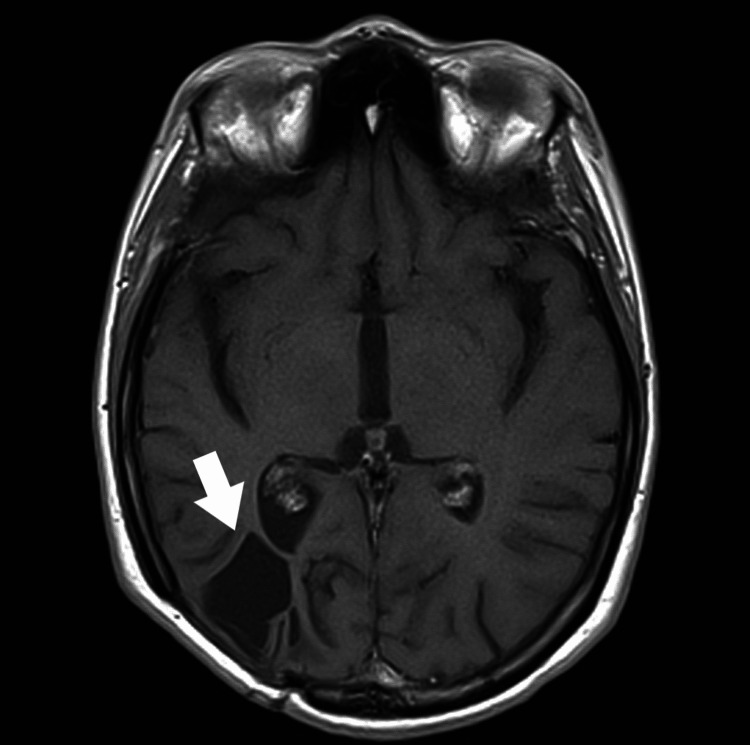
A follow-up MRI scan of the head three years after surgery: a top view. No signs of recurrence of the abscess (marked with a white arrow).

Similarly, no signs of abscess recurrence were found in another MRI of the brain performed during a follow-up three years after the operation (Figure 6).

The patient’s condition was stable. The patient remains under the care of the neurosurgery clinic. History, prompt diagnosis, and action are critical in the management of patients with BAs. Inflammatory changes of dental origin cannot be underestimated.

## Discussion

BAs are rare but potentially deadly conditions that have been reported to result from dental infections. Male patients are more at risk than women, with a mean age under 60 [13]. However, in one report, all studied cases of BAs of dental origin were found to occur in female patients, with a mean age of 34.8 years [1]. This fact may be linked to the presence of active foci in the oral cavity; this would increase the likelihood of odontogenic infections and hence be considered a risk factor for the development of BAs. In dental procedures, many cases of inflammation and periodontal diseases can result in bacteremia, especially when an injury is also present [3,14]. A study on non-traumatic BAs for which patients sought treatment over a period of 40 months [1] found half of the cases to be related to oral infection, based on clinical and bacteriological findings. This value is significantly higher than those noted in other retrospective studies, viz. 4% [15], 8.5% [3], 2% [15], and 1% [3], as well as in literature reviews. However, retrospective studies report that the studied BAs lacked any clear cause in 20%-30% of cases [1,14,16]. In most cases, it is usually not stated whether and how the focus of infection from the oral cavity was excluded. Mueller et al. [13] report that in all studied cases, the route of infection was hematogenous; none were of otogenic or sinusogenic origin, which may be attributed to prompt and vigorous treatment of ear and sinus infections.

Similarly, a study carried out over the period 2015 to 2019 found 57% of 55 cases of BA to be of odontogenic origin [3], and a thorough examination of radiographic materials revealed oral pathologies in 11 patients (25%) during hospitalization, in whom no predisposing infections were initially recorded. Only one patient had an unknown origin for the abscess, suggesting that some patients in previous studies [14-19] were misclassified. As oral pathological conditions do not always exhibit any clinical symptoms, dental and radiographic examinations during hospitalization are recommended. Eleven patients characterized as having BAs of odontogenic origin had only oral pathological conditions revealed in radiographic examinations (Rps) that were not assessed during hospitalization by a dental or maxillofacial surgeon [3]. Although panoramic radiographs have limited value in detecting certain oral pathological conditions compared to other radiographic techniques, they still allow for the detection of inflammatory conditions in the oral cavity in 20% of patients. Sinusitis was the second most common predisposing factor for infection, with periapical periodontitis or iatrogenic extrusion of foreign bodies into the maxillary sinuses potentially developing into odontogenic sinusitis, which requires combined dental treatment. As this condition may remain undiagnosed [17,18], it is recommended that patients with BAs and unilateral maxillary sinusitis should undergo an oral examination [19]. It should also be noted that diabetes has been suggested as a risk factor that may exacerbate odontogenic infections [20]. Individuals with diabetes and poor glycemic control may be at more risk of severe periodontal diseases, possibly due to problems with their immune system [5]. In odontogenic BAs, the course of the infection depends on the virulence of the bacteria, host resistance factors, and regional anatomy. As such, before antibiotics are administered, microbiological cultures should be performed to confirm odontogenic infection in the pathogenesis of BAs [16]: these samples should be taken from the oral cavity and the purulent discharge of the abscess. Antibiotic therapy kills a portion of the oral bacteria, thus preventing accurate identification of the colonies therein; however, rapid surgical intervention and preliminary antibiotic treatment are often required in life-threatening situations [1]. In such cases, it may not be possible to identify the source of infection. Nevertheless, the odontogenic source should always be considered, and efforts should be made to ensure oral hygiene is addressed. It should be emphasized that *Fusobacteria*, especially in odontogenic infections, often occur in mixed populations. Odontogenic and periodontal abscesses are an environment conducive to polymicrobial infections, and different species may dominate in different phases of the disease. *Fusobacterium gonidiaformans* may dominate in the early phase of infection (e.g., in the periodontium), and *Fusobacterium necrophorum* may be present from the beginning but only become apparent later, e.g., as a result of antibiotic selection or environmental changes in the abscess.

## Conclusions

Odontogenic infections are frequently associated with the formation of BAs, but distant lesions, e.g., in the oral cavity, can lead to complications. As such, their possibility must also be considered by the medical team. Treatment should be carried out using a multidisciplinary approach; the dental team should be involved at an early stage, allowing quick identification and treatment of possible odontogenic lesions to optimize clinical results. Always evaluate for periapical pathology in unexplained BAs, even in the absence of oral symptoms.

## References

[1] Carpenter J, Stapleton S, Holliman R (2007). Retrospective analysis of 49 cases of brain abscess and review of the literature. Eur J Clin Microbiol Infect Dis.

[2] Calfee DP, Wispelwey B (2000). Brain abscess. Semin Neurol.

[3] Jespersen FV, Hansen SU, Jensen SS (2023). Cerebral abscesses with odontogenic origin: a population-based cohort study. Clin Oral Investig.

[4] Kilian M, Chapple IL, Hannig M (2016). The oral microbiome - an update for oral healthcare professionals. Br Dent J.

[5] Forner L, Larsen T, Kilian M, Holmstrup P (2006). Incidence of bacteremia after chewing, tooth brushing and scaling in individuals with periodontal inflammation. J Clin Periodontol.

[6] Lu CH, Chang WN, Lin YC (2002). Bacterial brain abscess: microbiological features, epidemiological trends and therapeutic outcomes. QJM.

[7] Khandelwal N, Gupta V, Singh P (2011). Central nervous system fungal infections in tropics. Neuroimaging Clin N Am.

[8] Lee TH, Chang WN, Su TM (2007). Clinical features and predictive factors of intraventricular rupture in patients who have bacterial brain abscesses. J Neurol Neurosurg Psychiatry.

[9] Tseng JH, Tseng MY (2006). Brain abscess in 142 patients: factors influencing outcome and mortality. Surg Neurol.

[10] Mishra AK, Dufour H, Roche PH, Lonjon M, Raoult D, Fournier PE (2014). Molecular revolution in the diagnosis of microbial brain abscesses. Eur J Clin Microbiol Infect Dis.

[11] Cavuşoglu H, Kaya RA, Türkmenoglu ON, Colak I, Aydin Y (2008). Brain abscess: analysis of results in a series of 51 patients with a combined surgical and medical approach during an 11-year period. Neurosurg Focus.

[12] Da Silva AC, Viera PV, Bittencourt AA (2022). Brain abscesses due to odontogenic infection: case series. Spec Care Dentist.

[13] Mueller AA, Saldamli B, Stübinger S (2009). Oral bacterial cultures in nontraumatic brain abscesses: results of a first-line study. Oral Surg Oral Med Oral Pathol Oral Radiol Endod.

[14] Prasad KN, Mishra AM, Gupta D, Husain N, Husain M, Gupta RK (2006). Analysis of microbial etiology and mortality in patients with brain abscess. J Infect.

[15] Roche M, Humphreys H, Smyth E (2003). A twelve-year review of central nervous system bacterial abscesses; presentation and aetiology. Clin Microbiol Infect.

[16] Vidal F, Coutinho TM, Carvalho Ferreira D, Souza RC, Gonçalves LS (2017). Odontogenic sinusitis: a comprehensive review. Acta Odontol Scand.

[17] Bjørndal L, Amaloo C, Markvart M, Rud V, Qvortrup K, Stavnsbjerg C, Bjarnsholt T (2016). Maxillary sinus impaction of a core carrier causing sustained apical periodontitis, sinusitis, and nasal stenosis: a 3-year follow-up. J Endod.

[18] Ferguson M (2014). Rhinosinusitis in oral medicine and dentistry. Aust Dent J.

[19] Juncar M, Popa AR, Baciuţ MF, Juncar RI, Onisor-Gligor F, Bran S, Băciuţ G (2014). Evolution assessment of head and neck infections in diabetic patients--a case control study. J Craniomaxillofac Surg.

[20] Holmstrup P, Damgaard C, Olsen I, Klinge B, Flyvbjerg A, Nielsen CH, Hansen PR (2017). Comorbidity of periodontal disease: two sides of the same coin? An introduction for the clinician. J Oral Microbiol.

